# Case Report: Challenges of orthodontic treatment in patients with autism spectrum disorders diagnosed in adulthood

**DOI:** 10.3389/fpsyt.2025.1558789

**Published:** 2025-07-31

**Authors:** Motoko Watanabe, Chihiro Takao, Chizuko Maeda, Ikuo Yonemitsu, Yasuyuki Kimura, Risa Tominaga, Takahiko Nagamine, Takashi Ono, Akira Toyofuku

**Affiliations:** ^1^ Department of Psychosomatic Dentistry, Graduate School of Medical and Dental Sciences, Institute of Science, Tokyo, Tokyo, Japan; ^2^ Department of Orthodontic Science, Graduate School of Medical and Dental Sciences, Institute of Science Tokyo, Tokyo, Japan; ^3^ Department of Psychiatric Internal Medicine, Sunlight Brain Research Center, Yamaguchi, Japan

**Keywords:** autism spectrum disorder, orthodontic treatment, psychiatry, hypersensitivity, restricted, repetitive behaviors, adult patients, case report

## Abstract

Patients with autism spectrum disorders (ASDs) occasionally have difficulties in dental settings owing to specific features, including hypersensitivity and restrictive persistence. When features of ASD remain unnoticed until adulthood, dental procedures would be provided without considering the patient’s potential ASD traits. Herein, we present three cases of ASD diagnosed in adulthood that experienced difficulties during orthodontic treatment, resulting in unsatisfactory outcomes, and discuss the importance of planning treatment and management that takes their ASD features into consideration. Case 1 involved a 23-year-old man who complained of unstable occlusion for three years since the initiation of orthodontic treatment and required orthodontic retreatment to return to his previous dentition. Previous orthodontic treatments had been discontinued on some occasions because of the hospitalized pharmacotherapy for exacerbated psychiatric conditions. Case 2 involved an 18-year-old woman who complained of unbearable changes in dentition in her upper incisors and changed facial appearance during orthodontic treatment, which caused her to drop out of school. Case 3 involved a 41-year-old woman who complained of a sliding jaw, especially when wearing a retainer, and changes in facial appearance for five years following the alignment of her dentition. All cases experienced discomfort and exacerbation of psychiatric conditions that were diagnosed as ASD during orthodontic treatment. Their complaints of persistent discomfort, including intolerance to the changed occlusion or facial appearance, would relate to their features of ASD, including hypersensitivities and restricted and repetitive behaviors. A vicious cycle between the exacerbation of their psychiatric conditions and uncomfortable sensations would impede the satisfactory goals of orthodontic treatment. Indications for orthodontic treatment should be carefully discussed, and treatment management that considers the characteristics of ASD, especially those that were diagnosed in adulthood, is crucial. This case series highlights the necessity of multidisciplinary follow-up throughout the long-term orthodontic treatment period in this patient population.

## 1 Introduction

Patients with autism spectrum disorders (ASDs) occasionally encounter difficulties in dental settings because of specific features such as hypersensitivity, resistance to change, difficulty in social communication, and restricted and repetitive behaviors (1, 2). Specifically, their hypersensitivity to sounds, vibrations, tastes, smells, and other factors induced by general dental procedures may lead to a fear or flight response, and their resistance to change can cause a refusal of dental procedures, which is further complicated by communication difficulties. Additionally, patients with ASD have a higher rate of malocclusion, which may be related to their high prevalence of oral and dietary habits (3, 4).

Orthodontic treatments generally result in occlusal changes accompanied by temporal pain and discomfort, which sometimes negatively affect a patient’s psychological state, such as anxiety and depression, during its long treatment duration, even in patients without psychiatric comorbidities (5, 6). Managing patients with psychiatric comorbidities during orthodontic treatment is challenging (7, 8); however, the management of adult orthodontic treatment in patients with ASD has not been sufficiently discussed. Besides the increasing number of patients with ASD diagnosed in adulthood (9), the number of adult patients undergoing orthodontic treatment has also increased (10). In addition to psychological aspects, difficulties in terms of compliance and satisfaction have been reported (10). In some cases, the potential features of ASD are not significant during childhood but are diagnosed in adulthood. These specific features might be unnoticed by the dental staff and, as such, can slip through well-considered dental management.

Herein, we present cases of patients diagnosed with ASD in adulthood who showed various difficulties during orthodontic treatment, resulting in unsatisfactory outcomes. Based on their rarity and severity, these cases were selected from outpatients of our department. We also discuss the indications and crucial multidisciplinary management, addressing the gap between psychiatrists and orthodontists, for orthodontic treatment in patients with ASD diagnosed in adulthood.

## 2 Case description

Case 1: A 23-year-old man complained: “I cannot eat well nor sleep well because of an unstable occlusion that did not fit properly”. The patient required orthodontic retreatment to return to his previous dentition.

Orthodontic treatment was initiated three years prior. The patient retrospectively reported: “I wanted to reduce my appetite by orthodontic treatment but not align my dentition,” “I wanted to transform my body to become less socially reclusive,” and “I felt something uncomfortable soon after orthodontic treatment started but could not explain my feelings to the orthodontist”. The patient gradually became depressive and was hospitalized for pharmacotherapy, with diagnoses of ASD and co-occurring depression. His orthodontic treatment was discontinued for a year. The orthodontic treatment was restarted with a new orthodontist; however, it was suspended on several occasions by hospitalized pharmacotherapy because of exacerbation of his psychiatric condition, perception of self-inadequacy, inner unrest, and suicidal thoughts. Subsequently, the patient became concerned with the asymmetry of his teeth and extracted his upper left wisdom tooth, which reduced his concern. One year later, although the active treatment to align his dentition had been completed, his uncomfortable occlusal sensations persisted. The patient was referred to our department.

**Figure 1:**
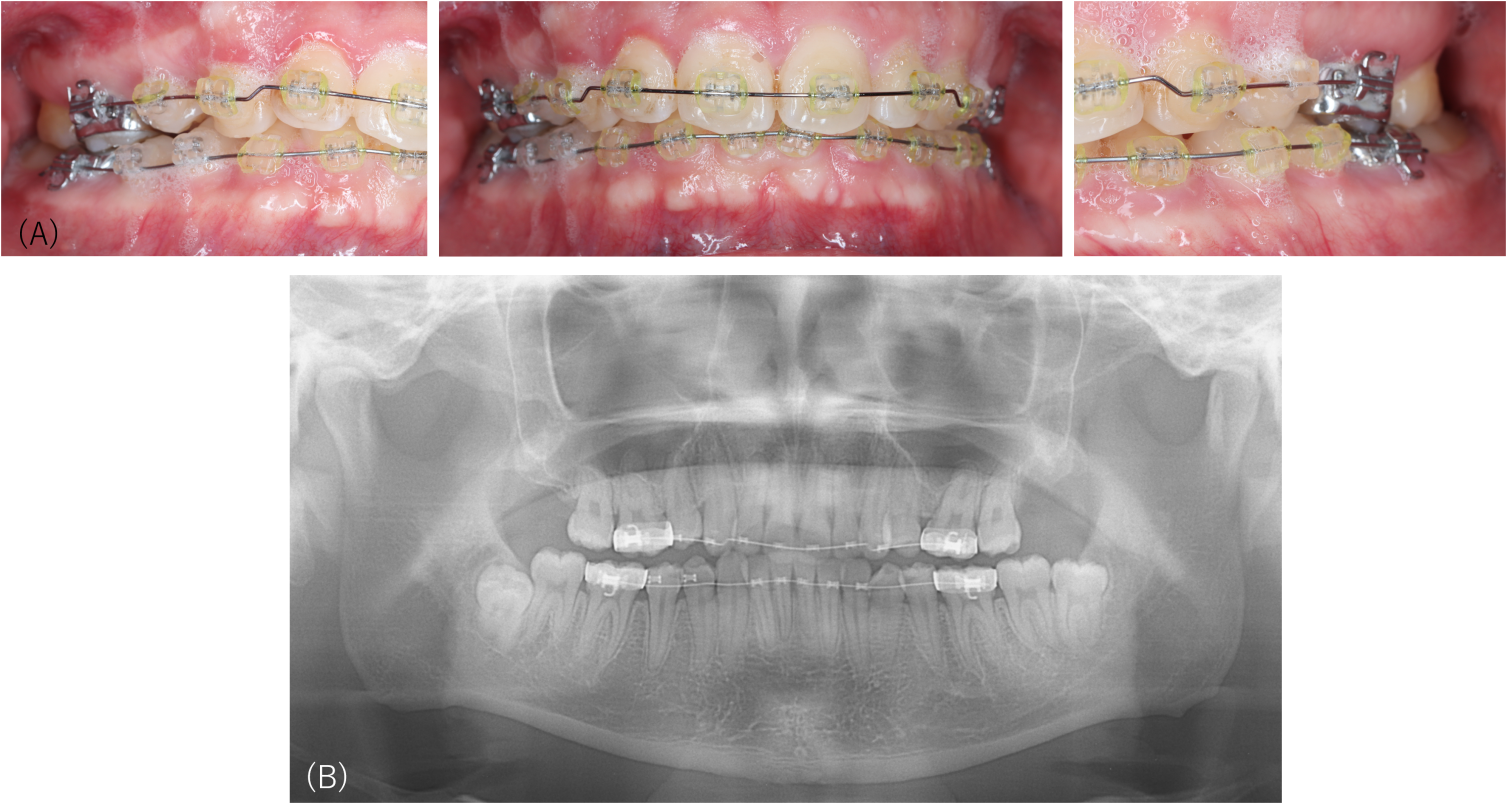
Intraoral pictures and the panoramic radiograph of Case 1. Occlusion of the molars is not orthodontically abnormal **(A)**, and no abnormal findings are detected by panoramic radiography **(B)** performed at the first examination at our department.

At the first examination at our department, the occlusion of the molars was not orthodontically abnormal (**Figure 1A**), and no abnormal findings were detected on the panoramic radiograph (**Figure 1B**). However, the patient requested orthodontic retreatment to regain his previous dental spacing followed by dental implant treatment to fill the space where the upper left premolar had been extracted. We, therefore, explained that providing dental procedures as he demanded was not suitable and would not resolve his concern. The patient requested to be hospitalized with his belief that modified electroconvulsive therapy would reduce his uncomfortable occlusal sensations. The procedure was discontinued shortly after because he did not achieve immediate improvement. The patient removed his orthodontic devices and visited different orthodontists to undergo treatments as he demanded.

Case 2: An 18-year-old woman complained of “difficulty making a natural smile without nervousness,” “an elongated face with tensioned muscles, and altered eyes,” and “difficulty in speaking”.

Orthodontic treatment was initiated three years prior, to align the upper incisors. No complication was observed until the beginning of orthodontic treatment with fixed appliances following the extraction of the upper first premolars and dentoalveolar expansion of the maxillary dentition. However, when the lingually dislocated upper lateral incisors moved labially, her complaints were induced and exacerbated until she discontinued the treatment after a few months. The patient gradually experienced difficulty attending high school even after moving to another institution. An esthetic dentist provided ceramic crowns on her upper incisors along with her demands, which, according to her report, reduced her sensations and made her entrance examination for university successful. However, the patient gradually had difficulty spending time at university. No previous psychiatric treatment was undertaken, except for her visiting a clinical psychologist who suspected her potential traits of ASD. Our department was asked to provide a second opinion on future orthodontic treatments.

**Figure 2:**
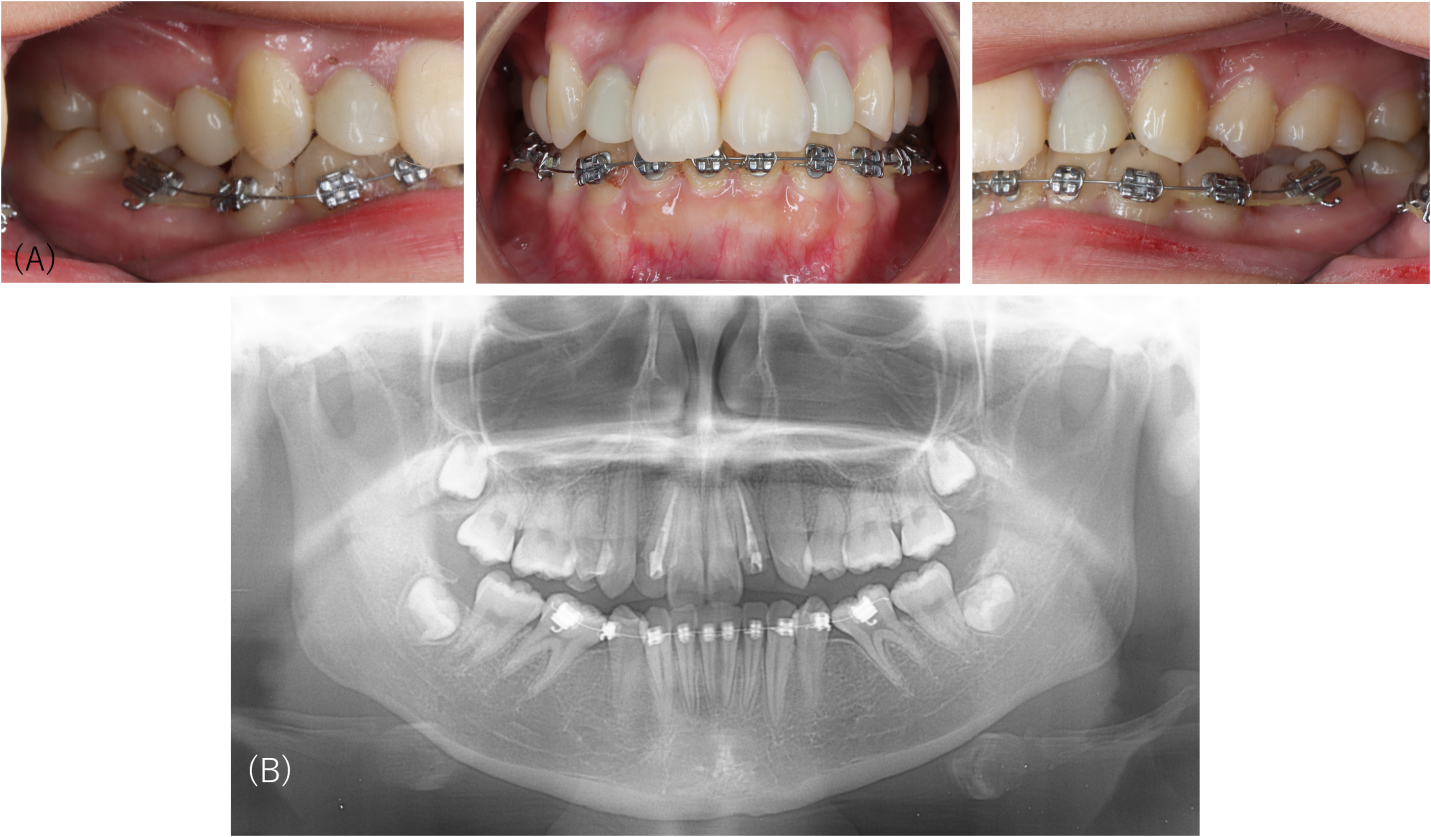
Intraoral pictures and the panoramic radiograph of Case 2. Intraoral examination and panoramic radiography reveal that the patient’s dentition is not aligned, which is an indication for orthodontic treatment.

Intraoral examination and panoramic radiography revealed that the patient’s dentition was not aligned (**Figure 2**) and could be an indication for orthodontic treatment; however, her psychiatric condition seemed unstable. Her unwell feeling made her unable to attend university; however, no continuous medical treatment had been provided. We suggested that the patient undergo a psychiatric assessment before restarting orthodontic treatment. The patient was referred to a psychiatrist, and pharmacotherapy was initiated with a diagnosis of ASD. The orthodontic treatment was discontinued, and the dental follow-up was subsequently lost.

Case 3: A 41-year-old woman complained that “My jaw is sliding and shifting like this…(showing that her mandible shifted horizontally)” and her facial appearance was with discomfort, “as if my mouth is not completely closed”.

**Figure 3:**
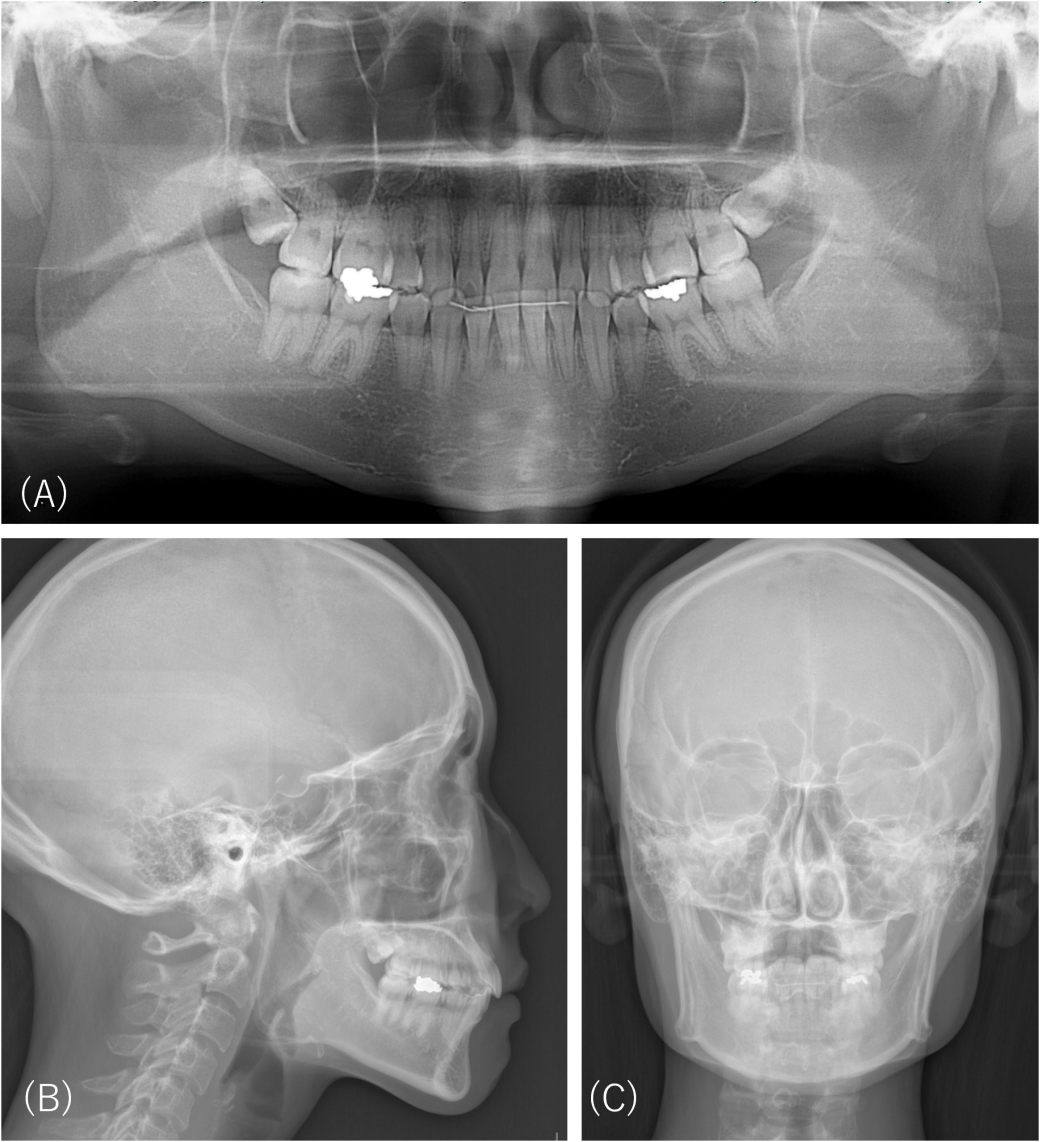
Radiographic images of Case 3 five years prior to the first examination at our department. There is no significant abnormality on the panoramic radiograph **(A)**, cephalograms of the facial profile **(B)**, or frontal view **(C)**.

Eleven years prior, orthodontic treatment was initiated. During the retention period after the six-year alignment of dentition, she began complaining of a sliding jaw and feeling as if her mouth was unclosed, which prevented her from wearing a retainer for a long time. The patient visited a psychiatrist because of unbearable orofacial discomforts and was diagnosed with ASD and body dysmorphic disorders. However, due to her refusal to undergo any psychiatric treatment, her follow-up was discontinued. At her first visit to the orthodontists at our university hospital, no significant abnormality was observed in the panoramic radiograph (**Figure 3A**), cephalograms of the facial profile (**Figure 3B**), or the frontal view (**Figure 3C**). Because the reversal correction after her orthodontic treatment was a minor crowding and the anterior facial height was not large, her dentition was deemed unsuitable for orthodontic retreatment. The orthodontist recommended myofunctional therapy for mouth closure rather than invasive dental procedures, including further orthodontic retreatment and orthognathic surgery. The patient refused this suggestion and repeatedly visited many orthodontists, oral surgeons, and plastic surgeons at other medical institutions for five years. None of the doctors she visited initiated orthodontic or plastic surgery procedures in line with the indication that her dentition and orofacial condition were within the normal range. The oral surgeon also noticed many old cut scars on her left wrist. The patient had trouble controlling her anger, specifically hurling her retainer by shouting and refusing to pay her doctor’s fees. The orthodontist referred the patient to our department for better management of her persistent orofacial complaints.

**Figure 4:**
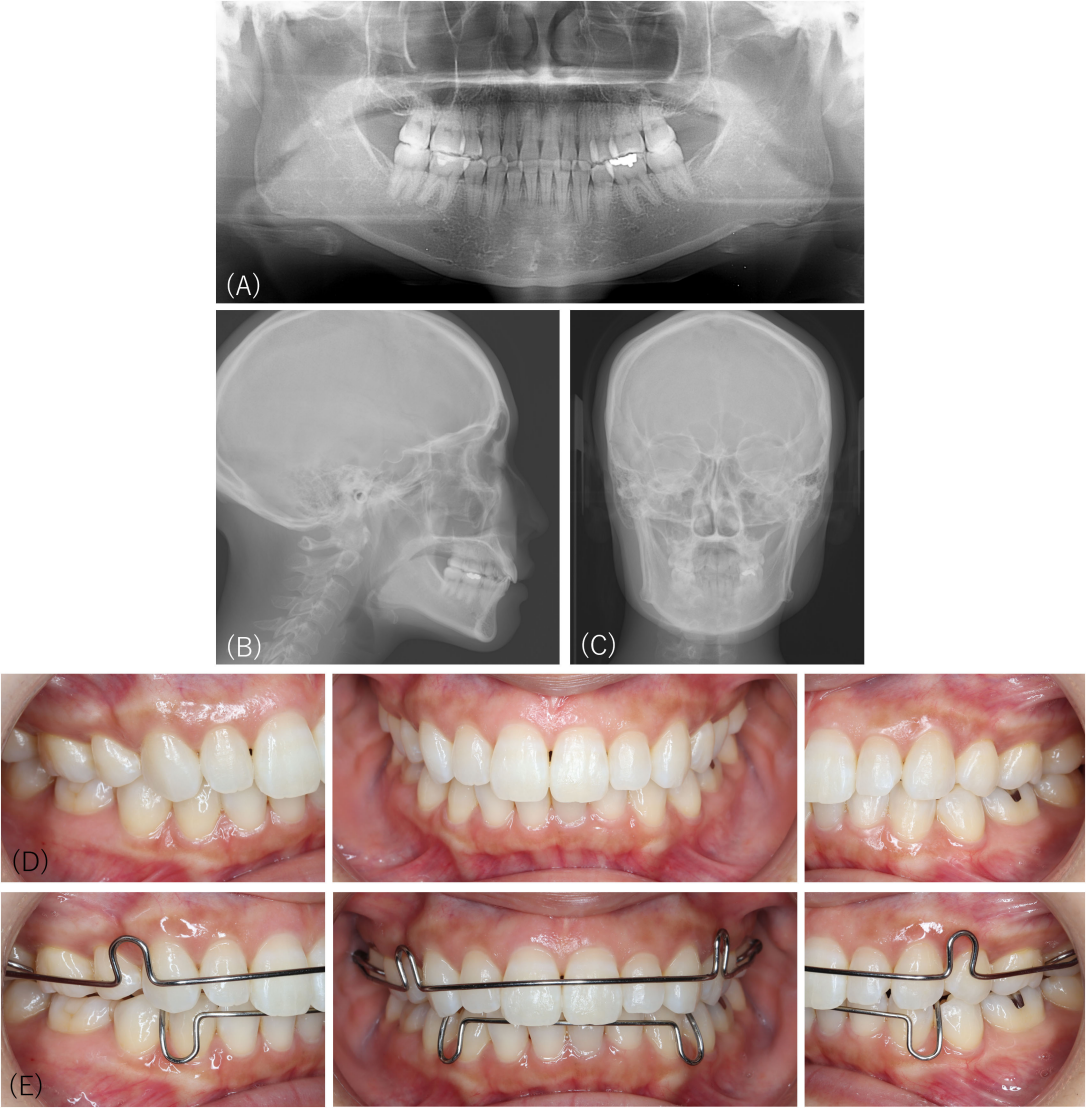
Radiographic images and intraoral pictures of Case 3 at the first examination at our department. There is no significant abnormality on the panoramic radiograph **(A)**, cephalograms of the facial profile **(B)**, or frontal view **(C)**. No changes are observed compared to previous radiographic examinations five years prior. There are no indications for orthodontic retreatment of the aligned occlusion **(D)**, although the patient slightly shifted her left mandible and complained of uncomfortable occlusion when wearing a retainer **(E)**.

At the first examination at our department, the findings in the panoramic radiograph, cephalograms of the facial profile, or frontal view were in the normal range (**Figures 4A–C**). No changes were observed compared to previous radiographic examinations five years prior (**Figure 3**). There were no indications for orthodontic retreatment of the aligned occlusion (**Figure 4D**), although the patient slightly shifted her left mandible and reported discomfort with her occlusion when wearing a retainer (**Figure 4E**). Her restricted concern about her facial appearance by describing her “sliding jaw,” and her irritability due to difficulties in communication through medical interviews were observed. We explained that her jaw and dentition were not suitable for orthodontic treatment; however, the patient did not accept this assessment but required orthognathic surgery to correct her facial appearance. Consultation with her psychiatrist revealed that she rarely visited her psychiatrist and did not recognize any remarkable difficulties in her daily life, although she experienced orofacial complaints. The patient visited the plastic surgeon, who, according to her report, informed her that her jaw was too large. The dental follow-up had been lost.

## 3 Discussion

This report presents patients who were diagnosed with ASD in adulthood and experienced difficulties during orthodontic treatments. One of the limitations of the present cases is that their orthodontic treatments were not satisfactorily completed. Meanwhile, this study highlights some considerable risks and the importance of multidisciplinary follow-up in this patient population.

The severity of ASD varies among individuals, and its characteristics may remain unnoticed until adulthood, similar to the cases in this report. Patients with ASD sometimes experience difficulties in dental settings because of features of ASD, such as hypersensitivity, unacceptability of change, and difficulty in communication (11). The present cases suggest that there are also some risks associated with adult orthodontic treatment for patients with potential traits of ASD.

First, hypersensitivity and restricted attention, which are specific features in patients with ASD, may induce difficulty in accepting changes in occlusions or facial appearance, even if the dentition is successfully aligned orthodontically. The patient in Case 1 could hardly accept aligned occlusion, despite the absence of occlusal abnormalities. Patients in Cases 2 and 3 could not accept their outlook in addition to the changed occlusions. All patients strongly demanded orthodontic retreatment despite our explanation that it was unnecessary. Their strict and detailed complaints may reflect hypersensitivity and restrictive and repetitive behaviors of ASD.

Second, their psychiatric conditions may be exacerbated during orthodontic treatment. Because patients with ASD often have other psychiatric diseases, such as depression and anxiety disorders (1), ASD and these comorbid conditions should be considered. The patient in Case 1 presented with worsening depression that co-occurred with ASD and discontinued orthodontic treatment several times. The patient in Case 2 exhibited difficulty adjusting to changes in social life during high school and university, while the patient in Case 3 struggled to control her emotions. All patients showed worsening psychiatric conditions in different ways. Even in patients without psychiatric comorbidities, long-term orthodontic treatment is generally associated with recurrent pain and discomfort. The research on adult orthodontic treatment has identified psychological impacts such as self-consciousness about wearing orthodontic devices, which can affect self-esteem and social interactions, treatment duration, and treatment compliance (10). Xie et al. reported that 56.3% of orthodontic patients, even without psychiatric comorbidities, presented with somatic pain, and 20.0% reported psychological discomfort, suggesting that psychological well-being is necessary for satisfactory orthodontic treatment (6). Especially in patients with ASD, psychological and somatic stress during orthodontic treatment may increase and exacerbate psychiatric conditions. The vicious cycle between unbearable discomfort relating to occlusal or facial changes by orthodontic treatment and worsening psychiatric conditions may cause patients to discontinue or prolong orthodontic treatment.

Difficulty in communication, another specific feature of ASD, can lead to misunderstandings between patients and orthodontists. Family support would be helpful; however, many patients with ASD diagnosed in adulthood visit orthodontists alone because they are adults and are not under parental guardianship. In addition to providing informative explanations, it is important to assess patients’ understanding of their condition and to identify their psychosocial challenges. When patients experience difficulties with emotional expression, practitioners should carefully observe their facial expressions and demeanor, talk to them, and confirm that they are not in distress. The goals of orthodontic treatment should be shared between patients and practitioners through interviews about their requirements for orthodontic treatment. The patient in Case 1 requested orthodontic treatment not to align his dentition but to change himself. The patients in Cases 2 and 3 required orthodontic retreatment, complaining of facial appearance rather than malocclusion. A previous study reported that some patients with psychiatric comorbidities required orthognathic surgery related to their psychiatric conditions and suggested the necessity for prudent consideration of the indications for orthodontic treatment, including orthognathic surgery (12). Clarifying patients’ expectations regarding what is attainable through orthodontic treatment is important. The practitioner should intervene when patients’ expectations are unreasonable. Sharing appropriate treatment goals may be essential to improving treatment satisfaction.

With the recent increase in the number of individuals with ASD in Japan (9), orthodontic patients with ASD may also increase. For patients with ASD, getting accustomed to visiting dentists and building good patient-dentist relationships from an early age has been suggested to improve oral health and satisfaction with orthodontic treatment outcomes (11). However, when patients are diagnosed with ASD in adulthood, as in the present cases, their features of ASD may be overlooked by the dental staff; hence, considerable support for dental treatments would be limited. Because all patients in this study experienced challenging symptoms and were dissatisfied with the results of orthodontic treatment, we recommend special consideration throughout orthodontic treatment, especially for patients diagnosed with ASD in adulthood.

The lack of common criteria for diagnosing ASD due to the assessment by each patient’s psychiatrist is another limitation in this report, besides uncompleted orthodontic treatment. However, the present cases suggest that problematic symptoms, including intolerance to the changed occlusion or facial appearance and exacerbation of psychiatric conditions relating to features of ASD, could make it difficult to achieve the satisfactory goal of orthodontic treatment in patients with ASD diagnosed in adulthood. Indications for orthodontic treatment should be discussed carefully, considering patients’ psychiatric conditions, especially when ASD is diagnosed in adulthood. Specific risks discussed above, as well as the advantages of orthodontic treatment, should be explained to patients before the initiation of orthodontic treatment. Furthermore, this case report highlights the importance of multidisciplinary follow-up throughout the long-term orthodontic treatment period. Establishing a team of orthodontists and psychiatrists is crucial for discussing the indications for orthodontic treatment and the type of specialized support that would be most beneficial for each patient. Most psychiatrists know little about the invasiveness of orthodontic treatment; similarly, most dentists hardly understand the difficulty of ASD in adulthood. Orthodontists can share their opinions with psychiatrists by presenting the potential risks associated with orthodontic treatment to address the gap between them. Further research is needed on management strategies for orthodontic patients with ASD diagnosed in adulthood.

## Data Availability

The original contributions presented in the study are included in the article. Further inquiries can be directed to the corresponding author.
